# Prevalence of overweight and obesity and associated factors among people living with HIV attending a tertiary care clinic in Uganda

**DOI:** 10.1186/s40795-022-00604-w

**Published:** 2022-09-27

**Authors:** Esther Alice Nalugga, Eva Laker, Maria Sarah Nabaggala, Ahmed Ddungu, Charles Batte, Theresa Piloya, Felix Bongomin

**Affiliations:** 1 Infectious Diseases Institute, Makerere University College of Health Sciences, Kampala, P.O. Box 22418, Uganda; 2 Lung Institute, Department of Medicine, Makerere University College of Health Sciences, Kampala, Uganda; 3 Climate and Health Unit, Tree Adoption Uganda (TAU), Kampala, Uganda; 4 Department of Paediatrics and Child Health, Makerere University College of Health Sciences, Kampala, Uganda; 5 Department of Medical Microbiology & Immunology, Faculty of Medicine, Gulu University, Gulu, P.O Box 166, Uganda; 6 Non-communicable and Infectious Diseases Research (NIDER) Platform, Kampala, Uganda

**Keywords:** Overweight, Obesity, Body mass index, Non-communicable diseases

## Abstract

**Background:**

Overweight and obesity are significantly increasing among people living with HIV (PLWH), contributing to the risk of major adverse cardio-metabolic events. However, little is known on its prevalence among PLWH in sub-Saharan Africa. In this study, we report the prevalence and factors associated with overweight and obesity among PLWH in a large tertiary HIV clinic in Kampala, Uganda.

**Methods:**

A cross-sectional, retrospective review of electronic database of all PLWH that attended the Adult Infectious Diseases Institute clinic between November 2018 and April 2019 was conducted. Demographic, body mass index (BMI) [kg/m^2^] and clinical variables were extracted. Based on BMI, nutritional status was classified as undernutrition (< 18.5kg/m^2^), normal (≥ 18.5 < 25kg/m^2^), overweight (≥ 25 < 30kg/m^2^) and obesity (≥ 30kg/m^2^). Poisson regression analysis was performed to determine factors associated with overweight and obesity.

**Results:**

Overall, 7,818 participants were included in the analysis, 64% (n = 4,976) were female, with a median age of 44 years (interquartile range (IQR): 36–51) and a median BMI of 24.2 (IQR: 21.2–28.1). The prevalence of overweight and obesity combined was 46% (55% female *versus* 30% male), obesity 18.2% (24.6% female *versus* 7.1% male) and overweight 27.8% (30.4% female *versus* 22.9% male). Factors associated with overweight and obesity were: Females (adjusted prevalence ratio [aPR]: 1. 8, 95%CI:1.69–1.87), age category 25—59 years (aPR: 1.9, 95%CI: 1.63–2.24) and ≥ 60 years (aPR: 1.8, 95%CI:1.49–2.12); duration on antiretroviral therapy (ART) for 6—10 years (aPR: 1.1, 95%CI:1.08–1.18), CD4 count 200–500 (aPR:0.08, 95%CI:0.01–0.15) and > 500 (aPR:0.46, 95%CI:0.39–0.54) and having at least one noncommunicable disease (NCD) (aPR: 1.1, 95%CI:1.07–1.18).

**Conclusion:**

There is a high burden of overweight and obesity among PLWH in Uganda. Nutrition and weight management programs particularly targeting high risk groups such as females and persons with underlying NCDs should be integrated into HIV care.

## Background

Human immunodeficiency virus (HIV) infection is usually associated with weight loss or undernutrition status [1–3]. Over the past two decades however, weight gain has been attributed to the increasing availability of highly active anti-retroviral therapy (ART) thus resulting into a marked decline in under-nutrition among people living with HIV (PLWH) [4]. On the contrary, overweight and obesity have been reported to be on the rise among PLWH in Africa [5]. Studies in some low income countries (LICs) show a higher burden of overweight and obesity among both ART-naïve (22.1%) [6] and ART experienced PLWH (34–35%) [7, 8] compared to under-nutrition (10.0–26.3%) [6, 9–13].

Weight gain in HIV is attributed to ART led immune reconstitution which prevents occurrence of several opportunistic infections [12] and leads to better health status. HIV is also associated with risk factors for overweight and obesity including poor dietary habits, alcohol intake and sedentary living [14, 15]. Overweight and obesity greatly pose an increased risk of non-communicable diseases (NCDs) like cardiovascular diseases, diabetes mellitus, dyslipidaemia, metabolic syndrome which complicate HIV management [16] and increase strain on health systems especially in resource limited countries like Uganda [17]. There is limited published information on the burden of overweight and obesity among the PLWH in Uganda. A study done in Kasangati Health centre IV, a peri-urban community clinic in Wakiso district in Uganda found the prevalence of overweight and obesity of 26% among PLWH [18].

An increasing trend of overweight and obesity was observed at the Infectious Diseases Institute (IDI) adult clinic, an HIV Centre of Excellence and tertiary HIV facility in Kampala, the capital city of Uganda. We sought to determine the prevalence and factors associated with overweight and obesity among PLWH attending IDI.

## Methods

### Study design

This was a cross-sectional, retrospective study conducted at the adult IDI Clinic from November 2018 to April 2019.

### Study setting

The study was conducted at the Infectious Diseases Institute Clinic, Makerere University College of Health Sciences. The clinic is located within Mulago National Referral hospital complex in Kampala, the capital city of Uganda. The clinic is PEPFAR funded and offers comprehensive HIV care and treatment to over 8,000 PLWH. IDI is a referral Centre for more complicated HIV/AIDS cases within the national referral system. The clinic runs several special clinics for different populations, such as people living with NCDs, sero-discordant couples, adolescents, elderly, pregnant mothers, most at risk populations (MARPs) and tuberculosis patients. Patients’ anthropometric measurements (weight, height, and computed BMI) are done by a nurse at triage and information entered into an electronic records system.

### Study population

All PLWH that visited the IDI Clinic from November 2018 to April 2019 were included. This period was sufficient to collect data of over 95% of the clients receiving care at the clinic and each had equal chance of being enrolled into the study. Eligible participants were all HIV- infected ART-experienced adults of both sexes aged ≥ 18years active in care at the IDI clinic (the most recent visit of each participant during the study period was considered). Patients with a missing height measurement, pregnant women, and those whose weight had not been taken within the last 6 months were excluded.

### Sample size

Sampling and sample size calculation was not done, as all eligible patients attending the IDI Clinic during the study period were included in the analyses. However, assuming binary covariates, with an estimated prevalence of overweight and obesity of about 26% from a previous study [18] among PLWH in Uganda, using formula of sample size calculation for a single population, with a margin of error of 5% at 95% confidence interval (CI), and a 80% power, a sample size of 296 participants with the primary outcome (overweight and obesity) was required. A power of 80% is the minimum required for detection of an effect size.

### Data collection procedures and measurements

Data was extracted by a review of patients’ electronic medical health records. The most recent clinic visit for each participant in the study period was considered. The maximum time taken by each patient to return to the clinic is 6months.Weight is routinely measured by a nurse on each patient’s visit while height is measured at the baseline visit, as part of routine HIV care, which include measuring height to the nearest 0.1cm using a stadiometer and weight to the nearest 0.1kg on a calibrated scale [19]. BMI, which is defined as the weight in kilograms divided by the square of the height in meters (kg/m^2^), is then calculated to assess the nutritional status of the individuals. BMI was classified according to the WHO as undernutrition ((< 18.5kg/m^2^), normal (18.5–24.9kg/m^2^), overweight (25–29.9kg/m^2^), and obesity (≥ 30kg/m^2^)[8].

### Dependent variable

The primary outcome in this study was the prevalence of overweight and obesity among the PLWH. Overweight was defined as a BMI of 25–29.9kg/m^2^ while obesity was defined as a BMI of ≥ 30kg/m^2^.

### Independent variables

We also extracted the following potential associated factors for overweight and obesity; age, sex, ART regimen, duration on ART, CD4^+^ T-cell counts, viral load and presence of NCDs. In Uganda, first line ART regimens consist of a combination of Tenofovir, Lamivudine and Dolutegravir or Efavirenz /Nevirapine. Second line therapy consists of either zidovudine or abacavir, lamivudine, and a boosted protease inhibitor. Third line agents are an optimised regimen based on drug-susceptibility testing, and in Uganda, boosted darunavir and dolutegravir are key components of the regimen.

### Data management and analysis

Information from the electronic database was verified with that from the patients’ charts and participants were excluded only if the required variable was missing in both the chart and the electronic system.

The descriptive statistics were presented with frequency counts and percentages for categorical variables. At bivariate analysis, modified Poisson regression was used because the prevalence of overweight and obesity was higher than 15%. The precision of the study was determined at 95% confidence interval (95%CI) with level of significance at p < 0.05. Prevalence ratio (PR) was used as the measure of association. At multivariable analysis, factors associated with the primary outcome at bivariable analysis were included in a multivariable model and modified Poisson regression model was done to estimate the adjusted prevalence ratio (aPR) and 95% CI. Analysis was performed using STATA version 13.0 (Stata Corp, college station, Texas, USA).

## Results

### Study recruitment

**Fig. 1:**
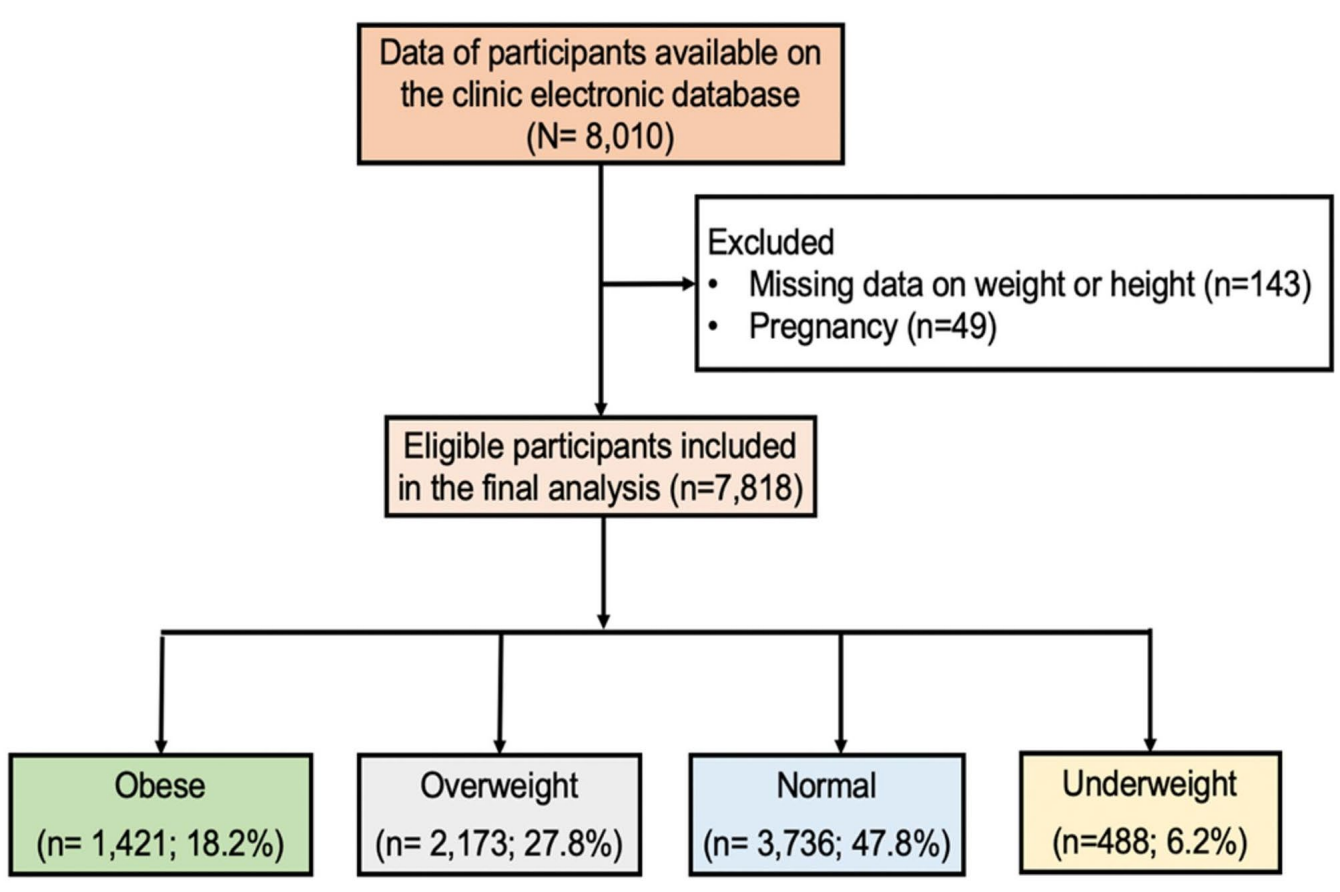
Study flow diagram

A total of 7,818 participants aged 18 years and older were included in the data analyses, we excluded 192 participants because of missing height or weight (143), or pregnancy (49), as shown in Fig.1.

### Characteristics of the study participants

Demographic characteristics of the 7,818 patients included in the analyses are shown in Table1. Among the participants, 64% (n = 4,976) were female, the median age was 44 years (IQR: 36–59 years) and 12.7% (n = 989) of the participants had at least one NCD (diabetes mellitus, hypertension, cancer, renal or heart disease).

**Table 1:** Characteristics among HIV-infected individuals at the Infectious Diseases Institute

	Variable	Number (%)
Sex		
	Female	4,976(63.65)
	Male	2,842(36.35)
Age, years		
	< 44	3987(51)
	≥ 44	3831(49)
Body mass index (kg/m^2^)		
	< 18.5	488 (6.24)
	18.5–24.9	3,736(47.79)
	≥ 25.0	3,594(45.97)
Duration on ART (years)		
	1–5	1,911(24.44)
	6–10	3,212(41.08)
	> 10	2,695(34.47)
CD4 + cell count(cells/mm^3^)	<200	1,610 (20.59)
	200 - <500	2,321(29.69)
	≥ 500	3,887(49.72)
Viral load count		
	Not suppressed(≥ 75copies)	1,236(15.81)
	Suppressed(< 75copies)	6,582(84.19)
ART regimen		
	First line	5,948(76.08)
	Second line	1,478(18.91)
	Third line/complex	392(5.01)
NCDs		
	None	6,829 (87.35)
	One or more Commonest NCDs categorized; Hypertension Diabetes Mellitus Renal disease	989 (12.65) 652 (8.3) 161 (2.1) 57 (0.7)

### Prevalence of overweight and obesity

**Fig. 2:**
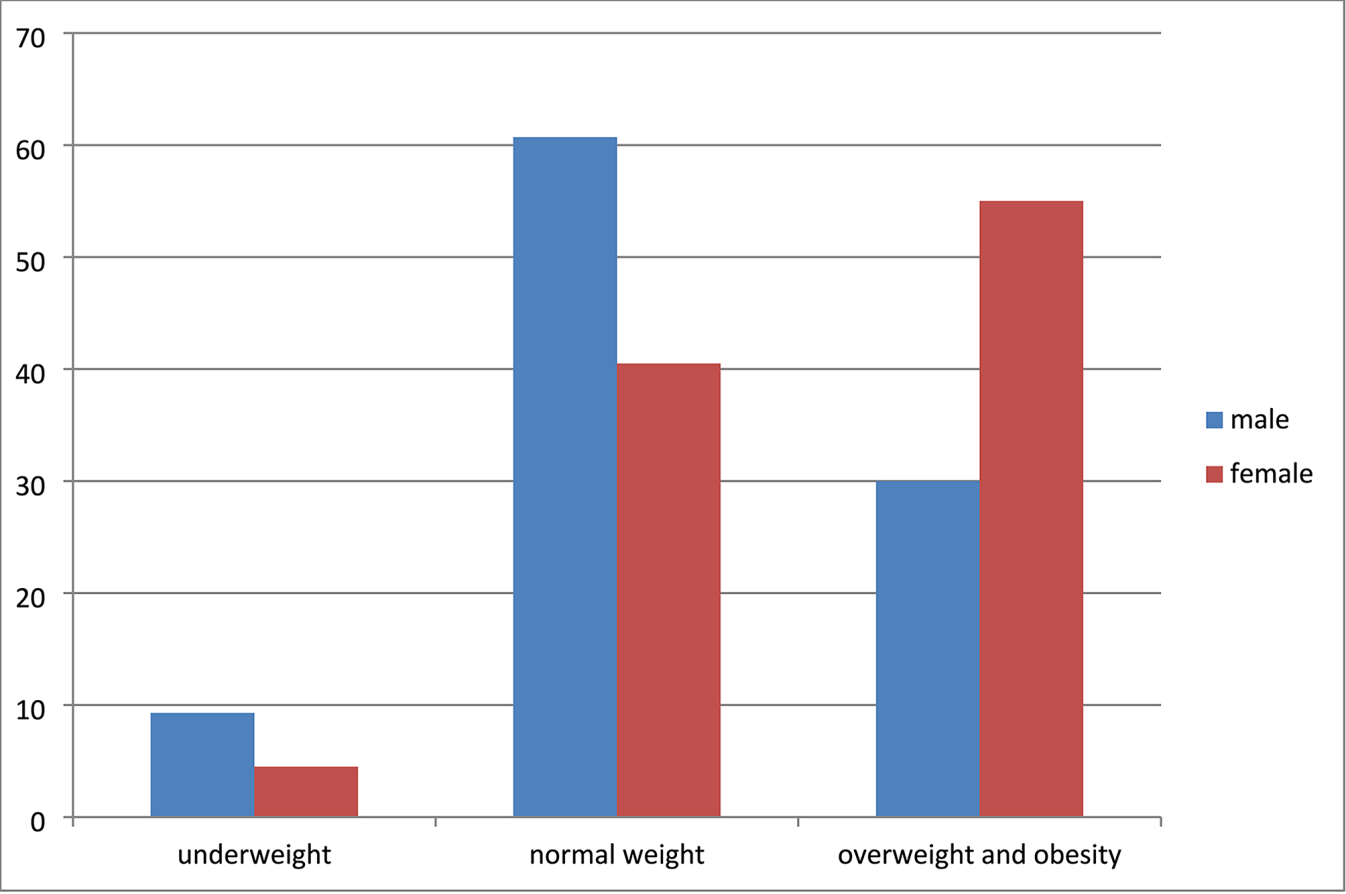
Prevalence of undernutrition, overweight and obesity by sex

The overall median BMI was 24.2kg/m^2^ (IQR = 21.2–28.1). The prevalence of overweight was 27.8% (IQR: 26.8–28.8) and obesity 18.2% (IQR: 17.4–19.1) among the overall participants in the clinic. The prevalence of both overweight and obesity was higher among women (55%) than men (30%) as illustrated in Fig.2. Similarly, the prevalence of obesity alone was higher among women (24.6%) than men (7.1%).

### Factors associated with overweight and obesity

Females, older age, longer duration on ART, CD4 + T-cell count and presence of NCDs were found to be associated with overweight and obesity (p < 0.05) (Table2).

**Table 2:** Univariable and multivariable associations of factors for overweight and obesity among HIV- infected individuals

Variables		Overweight & Obesity (BMI ≥ 25)			
		PR (95%CI)	P-value	aPR (95%CI)	P-value
Sex					
	Male	1.00		1.00	
	Female	1.86(1.77–1.95)	< 0.001	1.78(1.69–1.87)	**< 0.001**
Age					
	< 44	0.04 (0.002–0.08)	0.066	1.04 (0.99–1.07)	**< 0.001**
	≥ 44	1.00		1.00	
Duration on ART (years)					
	1–5	1.00		1.00	
	6–10	1.21(1.16–1.26)	< 0.001	1.13(1.08–1.18)	**< 0.001**
	> 10	1.00(0.95–1.05)	0.951	0.93(0.88–0.97)	**0.003**
CD4 Counts					
	<200	1.00		1.00	
	200–500	0.22(0.15–0.29)	< 0.001	0.08 (0.01–0.15)	**0.037**
	> 500	0.53(0.46–0.60)	< 0.001	0.46 (0.39–0.54)	**< 0.001**
ART Regimen					
	First line	1.00		1.00	
	s line	0.96(0.92–1.01)	0.092	1.00(0.96–1.05)	0.893
	Third line	0.96(0.89–1.03)	0.254	0.93(0.86-1.00)	0.052
Presence of NCDs					
	None	1.00		1.00	
	At least one	1.07(1.02–1.12)	0.008	1.12(1.07–1.18)	**< 0.001**
Viral load					
	Non-suppressed	1.00		1.00	
	suppressed	1.09(1.04–1.15)	< 0.001	1.02(0.97–1.07)	0.518

The probability of having overweight & obesity was almost 2-fold higher among female participants compared to males (aPR: 1.78, 95% CI:1.69–1.87). Compared to participants aged < 25 years, those aged 25–59 years had 1.9-fold (95% CI:1.63–2.24) and those aged ≥ 60 years had 1.8-fold (95% CI:1.49–2.12) higher risk of being overweight or obese. Compared to participants with CD4 counts < 500 cells/mm^3^, those with CD4 counts 200–500 cells/mm^3^ had 0.08-fold (95% CI: 0.01–0.15) and those with > 500 cells/mm^3^ had 0.46-fold (95% CI: 0.39–0.54) lower probability of being overweight or obese. The probability of overweight & obesity was 13% higher among participants whose duration on ART ranged from 6 to 10 years (aPR = 1.13; 95% CI: 1.08–1.18), but 7% lower among patients on ART for > 10 years (aPR = 0.93; 95% CI: 0.88–0.97), when compared to patients on ART for 1–5 years. The probability of overweight & obesity was 12% higher among patients with at least one NCD than those with none (aPR = 1.12; 95% CI: 1.07–1.18).

## Discussion

Our study findings show that the overall prevalence of overweight & obesity was 46% (overweight- 27.8% and obesity- 18.2%). This was slightly higher than the prevalence observed among 254 PLWH attending an ART clinic at Kasangati Health Centre IV, Uganda (overweight- 18.9% and obesity- 7.1%) [18]. Correspondingly, the Uganda NCD risk factor baseline survey found a prevalence of overweight at 14.5% and obesity 4.6% in the general population which was lower than our study findings [20].Our findings are however lower than those of a study done among 9,612 PLWH in KwaZulu-Natal, South Africa where prevalence of overweight was 45.7% and obesity 23.3% (58.7% in women and 15.9% in men) [9].

On the contrary, the prevalence of overweight & obesity combined was 10.6% in men and 22.6% in women in western Kenya [21], which was lower than that in our findings. Similarly, another study done among HIV patients in Tanzania found that the prevalence of overweight and obesity was 8% and 2% in men, and 16% and 7% in women, respectively [22], which was also lower than the prevalence observed among in our study. The prevalence of overweight & obesity surpassed that of undernutrition in the overall study population (46% vs. 6.2%). The same results were reported in a study done in Addis Ababa where prevalence of overweight & obesity was 22.1% compared to that of undernutrition which was 15.1% [6].

The reason for the high prevalence of overweight & obesity could be due to lifestyle changes which include poor dietary patterns and sedentary living which lead to increased weight gain [14]. Secondly, because wasting syndrome linked to AIDS is associated with stigma [23], PLWH tend to embrace weight gain through adoption of poor eating patterns like high caloric diets so as to avoid being stigmatised.

Our study findings show that overweight & obesity was almost twice in women than in men while obesity alone was thrice in women than in men. This was similar to the findings of the Uganda National NCDs risk factors STEPS survey where the prevalence of overweight & obesity was twice as much among the females than males [20]. Our findings were also comparable with other studies done among PLWH where overweight and obesity were more prevalent among females than males [6, 9, 21, 22]. This could be explained by the tendency for women to take up sedentary work or jobs with low physical demand thus increasing their risk of overweight & obesity as well as cultural values that favour larger body sizes as a sign of prosperity and responsibility [24, 25].

We also observed that overweight & obesity were more prevalent among the older patients, with the middle-aged adults (25–59 years) having the highest risk. This was similarly observed in the study done in Tanzania where older patients were more likely to be overweight or obese[22]. This we presume to be due to the decrease in physical activity as well as the decreased metabolism associated with aging [26].

In our findings, duration of ART ranging from 6 to 10 years was positively associated with overweight & obesity. Interestingly, duration of more than ten years on ART was negatively associated with overweight and obesity. This we hypothesised to possibly be due to lipoatrophy caused by drugs like stavudine (d4T) which were used in ART regimens in the earlier days of HIV management [27].

Our findings also showed that patients that had at least one NCD were more likely to have overweight and obesity. Evidence shows that overweight and obesity are some of the known cardio-metabolic risks for NCDs [15]. On the contrary, presence of some NCDs is likely to limit physical activity. Similar findings were observed in other studies done among HIV patients in Kenya and South Africa [9, 21]. Our findings highlight the imminent need to create awareness among health workers and policy makers in designing of nutrition care packages which include weight management programs, prevention of NCDs as well as act as a baseline for larger studies on nutrition in HIV/AIDS.

There were some limitations in conducting this study; this was a retrospective analysis of routinely collected clinical data therefore some variables of interest were not routinely measured for example dietary intake, level of physical activity, tobacco use, alcohol use, education level and employment status among others. Secondly, it is not possible to determine causal relationships between the risk and outcome variables.

## Conclusion

In conclusion, this study showed that there is an existing high burden of overweight and obesity among PLWH and this is more prevalent among females. Targeted nutrition education and weight management programs should be integrated into routine HIV care so as to curb the levels of overweight and obesity. It is also necessary to routinely screen HIV patients for NCDs. Additionally, further research is required to establish factors responsible for this drastic increase in overweight and obesity in HIV and remedies on how to combat this problem.

## Data Availability

The datasets generated and/or analysed during the current study are not publicly available due to potential breach of confidentiality but are available from the corresponding author on reasonable request.
